# Breast Metastasis From Colorectal Carcinoma Identified by Gene Assay: Case Report and Review of Literature

**DOI:** 10.7759/cureus.54952

**Published:** 2024-02-26

**Authors:** Sugam Gouli, Ansu Karki, Jeffery Allerton

**Affiliations:** 1 Hospital Medicine, Rochester Regional Health, New York, USA; 2 Internal Medicine, Bassett Medical Center, Cooperstown, USA; 3 Hematology / Oncology, Bassett Medical Center, Cooperstown, USA

**Keywords:** diagnostic challenge, immunohistochemistry, cancertype id, breast metastasis, colorectal cancer

## Abstract

Colon cancer metastasis to the breast is a rare presentation and has a poor prognosis. Diagnosis of the primary site for the metastasis is usually aided by immunohistochemistry but can be non-conclusive. Gene expression assay can be helpful in diagnosing the primary cancer site.

This is a 67-year-old female who presented with breast cancer metastasis of colorectal carcinoma origin. Immunohistochemistry was positive for cytokeratin AE1/AE3 cocktail and negative for ER, PR, CK20, CK7, CDX-2, and GATA-3, which was non-specific for any site of origin. The primary site was later identified using the gene expression assay CancerTYPE ID (BioTheranostics, Inc., San Diego, CA) which suggested likely primary was colon adenocarcinoma.

This case showcases a rare presentation of colon cancer metastasis to the breasts and how gene expression assay can help us find the primary origin of cancer metastasis when we have an unclear diagnosis from immunohistochemistry.

## Introduction

Colorectal cancer is the third most common cancer among both males and females in the United States. The incidence of colorectal cancer is increasing because of dietary practices and increased cancer screening. The most common sites of metastasis are the liver, bone, and brain. Colon cancer metastasis to the breast is a rare presentation. Usually, the identification of primary malignancy with breast metastasis is based on the patient’s history, clinical examination, radiological features, the morphology of the tumor and immunohistochemistry (IHC). IHC aids in the diagnosis of the primary site but sometimes can be non-conclusive. Gene expression assay, which detects the protein from abnormally functioning genes in genetically altered cancer cells, can help establish the primary site in such cases. Here, we present a case of colorectal carcinoma metastasis to the breast and how a gene expression assay helped detect the primary site of origin as colorectal carcinoma.

## Case presentation

**Figure 1:**
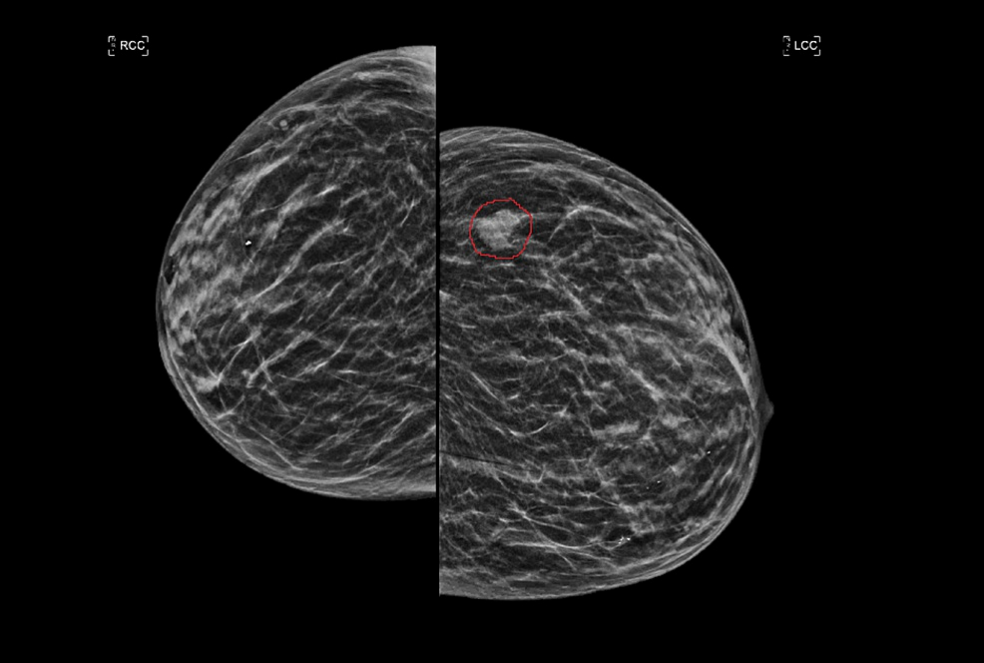
Left breast mammogram showing new lobulated lesion measuring 9 x 11 mm located in the left upper outer quadrant (red circle)

**Figure 2:**
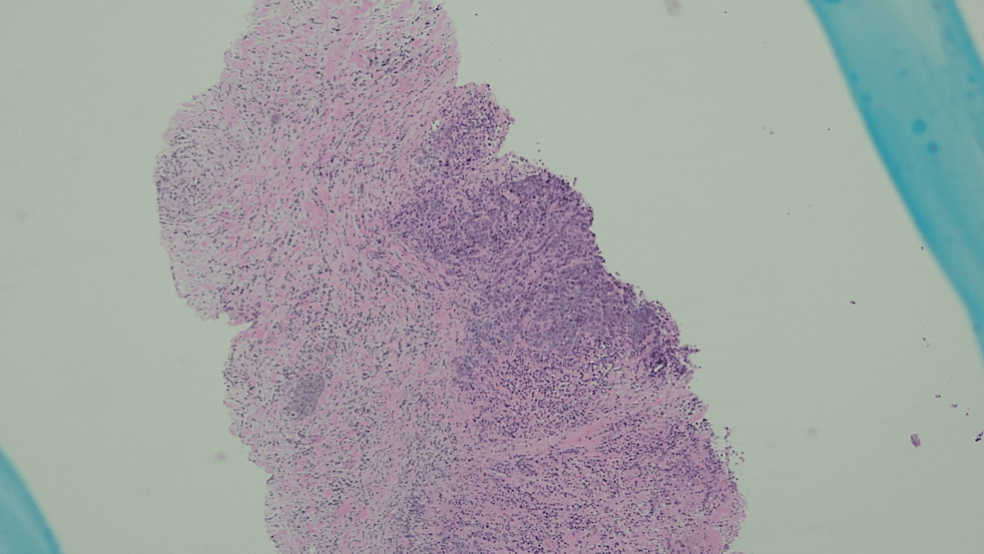
Histopathology of breast tissue showing tumor cells (bluish areas) with extensive desmoplasia; the replacement of normal tissue by fibrosis can also be seen (hematoxylin and eosin stain, 10x magnification)

**Figure 3:**
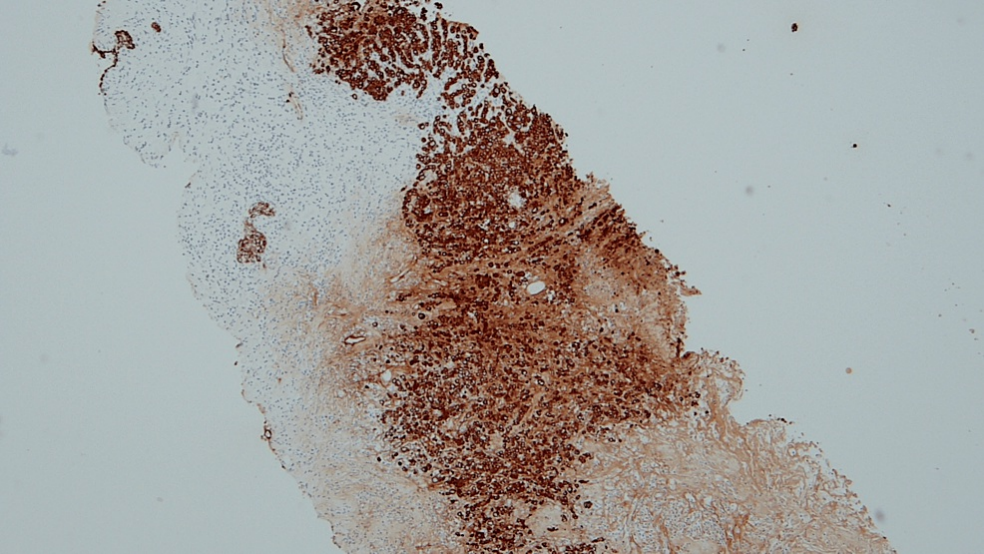
Immunohistochemistry showing breast core biopsy tissue positive for cytokeratin AE1AE3 cocktail (10x magnification)

**Figure 4:**
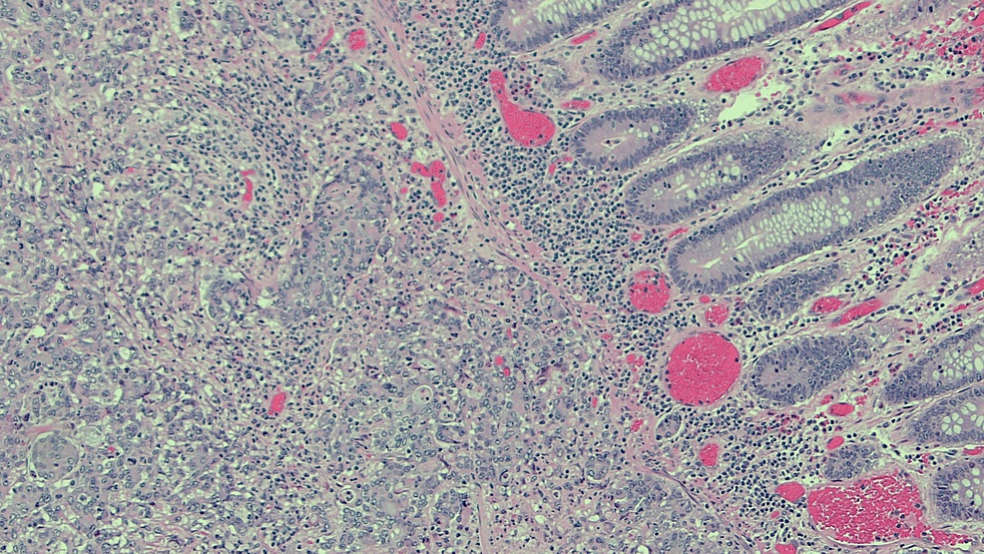
Histopathology of the colon with overlying normal mucosa and infiltrative tumor in the submucosa (hematoxylin and eosin Stain, 100× magnification)

A 67-year-old female presented with progressive right lower quadrant abdominal pain/cramps, constipation with intermittent loose stool. CT abdomen and pelvis was done which revealed a mass lesion in the ascending colon. She subsequently underwent a colonoscopy which revealed a large ulcerative mass at the hepatic flexure, with pathology demonstrating a poorly differentiated adenocarcinoma. She underwent laparoscopic right hemicolectomy with a final diagnosis of stage IIIB, pT3, pN1b, cM0, G3, and poorly differentiated adenocarcinoma of the hepatic flexure. She was started on adjuvant chemotherapy with folinic acid, fluorouracil, and oxaliplatin (FOLFOX). Subsequently, on a routine mammogram, she was found to have an irregular hypoechoic left breast mass at 2:00, approximately 7 cm from the nipple, measuring 1.4 x 0.8 cm (Figure 1). US-guided left breast biopsy revealed poorly differentiated adenocarcinoma (Figure 2) and further immunohistochemical analysis was done on the breast specimen in an attempt to further clarify the site of origin. It was positive for Ki67 40%, p53 +50%; positive for cytokeratin AE1/AE3 cocktail, and negative for ER, PR, CK20, CK7, CDX-2, and GATA-3 (Figure 3). It was not specific for a particular site of origin. Given the history of colon cancer, prior slides were reviewed (Figure 4). The prior colon tumor was poorly differentiated with areas of necrosis and some areas lacking gland formation, which was morphologically similar to the current left breast tumor metastasis.

The patient also developed new left inguinal lymphadenopathy after the first cycle of adjuvant chemotherapy and histopathology showed poorly differentiated carcinoma with zonal necrosis. Immunohistochemistry supported colonic origin. Gene assay (CancerTYPE ID, BioTheranostics, Inc., San Diego, CA) was performed on both breast and lymph node specimens. The lymph node showed “90% gastroesophageal adenocarcinoma and 6% intestinal adenocarcinoma” and the left breast specimen showed “82% intestinal adenocarcinoma, 14% gastroesophageal adenocarcinoma, and 95% not breast cancer”. The patient underwent left partial mastectomy with sentinel lymph node biopsy following three cycles of FOLFOX chemotherapy. The resected specimen histopathology showed central necrosis surrounded by dense fibroblastic reaction and chronic inflammatory cell infiltration mixed with macrophages with no evidence of DCIS, or lymphatic /vascular invasion.

## Discussion

Both breast cancer and colorectal cancer are the most common types of cancer, breast cancer being the most common cancer in the female population. At the time of diagnosis, about 6% of breast cancer are from metastatic breast cancer [1]. Breast metastasis is mostly from the ovary, lung, skin, and stomach. Colorectal carcinoma (CRC) is the third most commonly diagnosed carcinoma and approximately 15-25% of all CRC patients have distant metastasis but CRC metastasis to the breast is quite rare [2]. McIntosh et al. reported the first case of CRC with breast metastasis in 1976 [3]. In a literature review, we identified several case reports (40+) with breast metastasis from CRC. Hsieh and Hsu conducted a systematic review where 46 cases of CRC were identified, with a median age of 52 years. Among them, 10 cases had an average time of diagnosis from CRC to breast cancer metastasis of 28 months, a mean survival period was 14.9 months, and a five-year survival rate was 13.8% [4]. Hasegawa et al. identified 24 cases of CRC, out of which three patients died within six months (0.5-16 months) after diagnosis of breast metastasis [5]. Another review by Zhang et al. identified 32 cases with a median age of 51.1 years and an interval time from diagnosis of CRC to breast cancer of 25 months; five cases had an average survival of 14.5 months [6].

Immunohistochemistry (IHC) plays an important role in confirming the primary site. As per Bayrak et al., the CK7-/ck20+ phenotype is a very important marker, with 65.8% of patients having that mutation associated with colorectal cancer [7]. Any breast mass that is negative for all breast markers (ER, PR, Her2, GCDP15, BCA, and CK7) and positive for CK20 and CDX2 can be considered as a metastasis from the colon [8]. Most metastatic breast cancer cases can have a delay in diagnosis because of inconclusive IHC. IHC results in our patient showed CK7(-), ER(-), and PR(-) which meant the absence of breast carcinoma, however, CK20 and CDX2 were also negative which was inconclusive of colorectal origin, thus posing a dilemma.

CancerTYPE ID is a commercialized 92-gene cancer classification assay that has shown a sensitivity of 87.8%, favorable to the histopathological method [9]. CancerTYPE ID was sent for our patient, revealing an intestinal origin, and thus hinting towards the CRC as the primary origin for breast metastasis. It can thus be helpful in difficult diagnoses of malignancy found in unexpected locations like in our case or those with poorly diagnosed morphology/IHC as in in our patient.

## Conclusions

Colorectal cancer metastasis to breast is rare and prognosis is poor because of few months of median survival time. Proper identification of primary site can be done by combination of clinical history, imaging, pathology and immunohistochemistry. For cases that have diagnostic ambiguity even after IHC testing, genetic molecular assay can help with the final diagnosis of primary cancer in cancer metastasis.
